# From advanced imaging to molecular insights: the state of the art in omics for axial spondyloarthritis

**DOI:** 10.1177/1759720X251398430

**Published:** 2025-12-17

**Authors:** Iván Arias-de la Rosa, Jesús Eduardo Martín-Salazar, Laura Cuesta-López, Clementina López-Medina C, Alejandro Escudero-Contreras, Eduardo Collantes-Estévez, Nuria Barbarroja, María Ángeles Puche-Larrubia

**Affiliations:** Rheumatology Service/Department of Medical and Surgical Sciences, Maimonides Institute for Research in Biomedicine of Cordoba/University of Cordoba/Reina Sofia University Hospital, GC-05 Group, Second Floor, IMIBIC, Avda. Menéndez Pidal s/n, 14004 Córdoba, Spain; Department of Gastroenterology, Hospital General de Tomelloso, Tomelloso, Spain; Instituto de Investigación Sanitaria de Castilla-La Mancha, Toledo, Spain; Centro de Investigación Biomédica en Red de Enfermedades Hepáticas y Digestivas, Toledo, Spain; Rheumatology Service/Department of Medical and Surgical Sciences, Maimonides Institute for Research in Biomedicine of Cordoba/University of Cordoba/Reina Sofia University Hospital, Córdoba, Spain; Rheumatology Service/Department of Medical and Surgical Sciences, Maimonides Institute for Research in Biomedicine of Cordoba/University of Cordoba/Reina Sofia University Hospital, Córdoba, Spain; Rheumatology Service/Department of Medical and Surgical Sciences, Maimonides Institute for Research in Biomedicine of Cordoba/University of Cordoba/Reina Sofia University Hospital, Córdoba, Spain; Rheumatology Service/Department of Medical and Surgical Sciences, Maimonides Institute for Research in Biomedicine of Cordoba/University of Cordoba/Reina Sofia University Hospital, Córdoba, Spain; Rheumatology Service/Department of Medical and Surgical Sciences, Maimonides Institute for Research in Biomedicine of Cordoba/University of Cordoba/Reina Sofia University Hospital, Córdoba, Spain; Rheumatology Service/Department of Medical and Surgical Sciences, Maimonides Institute for Research in Biomedicine of Cordoba/University of Cordoba/Reina Sofia University Hospital, Córdoba, Spain; Cobiomic Bioscience SL, Córdoba, Spain; Rheumatology Service/Department of Medical and Surgical Sciences, Maimonides Institute for Research in Biomedicine of Cordoba/University of Cordoba/Reina Sofia University Hospital, Av. Menéndez Pidal, s/n, Poniente Sur, 14004 Córdoba, Spain

**Keywords:** artificial intelligence, axial spondyloarthritis, biomarkers, imaging, omics, precision medicine

## Abstract

Axial spondyloarthritis (axSpA) is a chronic inflammatory disease characterized by a complex interplay of molecular factors. Despite advances in understanding its pathophysiology, diagnostic delays and the absence of personalized treatment strategies remain significant challenges. This review provides a comprehensive analysis of imaging techniques and molecular approaches to improve disease characterization. Advanced imaging methods, including magnetic resonance imaging, positron emission tomography, and artificial intelligence (AI)-driven models, have enhanced diagnostic accuracy, reduced variability in interpretation, and facilitated early disease detection. In parallel, omics technologies have provided valuable insights into disease pathogenesis. Genomic studies have identified susceptibility loci beyond human leukocyte antigen B27, implicating key immune pathways such as interleukin-23/interleukin-17 signaling. Epigenomic modifications, particularly DNA methylation, play a key role in regulating gene expression in immune cells, especially within genetically predisposed loci. Transcriptomic studies have uncovered dysregulated immune pathways and revealed novel cellular players in disease pathogenesis, including CD99^+^ neutrophils, natural killer cells, and microRNAs—important post-transcriptional regulators that have shown high diagnostic accuracy when assessed in peripheral blood mononuclear cells. Proteomic analyses have further contributed by identifying potential biomarkers and therapeutic targets in blood using advanced technologies, highlighting molecules such as tumor necrosis factor, FK506-binding protein-like (FKBPL), mitogen-activated protein kinase 14 (MAPK14), interleukin 7 receptor, and interleukin-23 receptor, among others. Future research should focus on combining multi-omics data with AI-driven approaches to improve biomarker discovery, optimize patient classification, and guide personalized treatments. Bridging the gap between molecular insights and clinical applications will enable precision medicine strategies, improving early diagnosis and therapeutic outcomes in axSpA.

## Plain language summary

Understanding axial spondyloarthritis: how imaging and molecular tools can help diagnose and treat the disease earlier

Axial spondyloarthritis (axSpA) is a chronic condition that causes inflammation in the spine and pelvis, leading to pain, stiffness, and long-term disability. It often takes years to diagnose, and many patients do not receive treatments tailored to their specific disease features. In this article, we explain how modern imaging and molecular techniques can help improve how this disease is detected and managed. New imaging technologies, such as MRI (magnetic resonance imaging), PET scans (positron emission tomography), and computer-based tools using artificial intelligence (AI), are making it easier to detect early signs of inflammation and damage. These tools also help doctors make more consistent and accurate diagnoses. At the same time, scientists are using advanced laboratory techniques called omics to study the disease at the molecular level. These include: Genomics, which looks at DNA changes that may increase the risk of developing axSpA. Epigenomics, which explores how genes are turned on or off, especially in immune cells. Transcriptomics, which investigates how immune cells behave and which genes are active in them. Proteomics, which focuses on the proteins circulating in blood that may serve as warning signs or targets for treatment. Researchers have found several promising molecules and immune pathways that may help identify the disease earlier and create more precise treatments. Looking ahead, combining all these types of data along with the help of AI could allow doctors to better group patients based on their disease characteristics and offer personalized therapies. This could improve the lives of people with axSpA by reducing delays in diagnosis and making treatments more effective.

## Introduction

Spondyloarthritis (SpA) refers to a heterogeneous group of chronic inflammatory diseases characterized by the involvement of the axial skeleton, peripheral joints, and extra musculoskeletal features, including psoriasis, uveitis, and inflammatory bowel disease (IBD). SpA patients have traditionally been classified into subtypes based on this peripheral and/or extra-musculoskeletal manifestations, such as ankylosing spondylitis, psoriatic arthritis, IBD-associated SpA, reactive arthritis, juvenile SpA, and undifferentiated SpA.^1^ The key feature of these conditions is their shared clinical, radiological, genetic, and therapeutic characteristics, suggesting a common underlying pathophysiological mechanism strongly associated with human leukocyte antigen (HLA) B-27.^2^ In terms of classification, the Assessment of SpondyloArthritis International Society (ASAS) developed and validated criteria for axial spondyloarthritis (axSpA) in 2009^3^ and, more recently, issued a consensus definition of early axSpA in 2024.^4^ While nosology remains debated—that is, whether SpA subtypes reflect a spectrum of one disease or distinct entities with overlap—these ASAS updates refine disease boundaries and improve comparability across studies.^3,4^ Given the diversity of SpA phenotypes, this review focuses exclusively on axSpA. This decision stems from the considerable diagnostic and therapeutic challenges that uniquely characterize axSpA compared to other SpA subtypes. axSpA, encompassing both radiographic and non-radiographic forms, is associated with a substantial diagnostic delay, with a global average of 6.7 years.^5^ This can result in a significant disease burden, encompassing increased disease activity and impaired function.^5^ Time to diagnosis in axSpA is multifactorial, partly due to the complexity of diagnosis. In addition to delays, misdiagnosis and overdiagnosis are frequent, with the latter leading to unnecessary treatments, increased healthcare costs, and patient anxiety.^6,7^ Despite therapeutic advances, selecting the most effective treatment for each patient remains challenging. The ability to predict treatment response and disease progression would enhance therapeutic management.^8^

Omics technologies, including genomics, epigenomics, transcriptomics, proteomics, and metabolomics, offer promising tools for improving diagnosis, risk stratification, and treatment of axSpA. By identifying biomarkers linked to disease susceptibility, progression, and therapy response, they support precision medicine. For this narrative review, we conducted a structured search of PubMed/MEDLINE covering the period from 1994 to 2025. Search terms combined disease (“axial spondyloarthritis” or “axSpA”) with imaging modalities (“radiography” OR “X-ray” OR “MRI” OR “low-dose CT/LDCT” OR “PET/CT”), molecular layers (“genomics” OR “epigenomics” OR “transcriptomics” OR “proteomics”), and analytic approaches (“artificial intelligence” OR “deep learning” OR “radiomics”). We included peer-reviewed human studies in English (original research and reviews) addressing imaging, multi-omics, or artificial intelligence (AI) in axSpA. When multiple sources addressed the same question, we prioritized higher-impact journals, multicenter cohorts, and guideline or consensus statements. In addition, we deliberately retained a small number of seminal references (e.g., foundational genetic studies and early classification or imaging frameworks) because they provided the original descriptions of concepts and methodologies that remain essential in current research and clinical practice. To ensure balance and currency, these seminal works were systematically paired with recent publications (2019–2025) that validate or refine their conclusions. The integrated analysis provides a comprehensive view of disease mechanisms and biomarker discovery, paving the way for future translational research and clinical application.

## Concept of precision medicine

Precision medicine aims to optimize therapy by classifying individuals into subgroups based on disease susceptibility, prognosis, or treatment response. This approach integrates genomic, biomarker, phenotypic, and psychosocial data to guide clinical decision-making.^9^

Although often used interchangeably, precision medicine and personalized medicine are not synonymous. The first one is the preferred term, as it emphasizes the use of large-scale biological and clinical data to tailor treatments to defined patient subgroups. By contrast, personalized medicine may misleadingly suggest individually customized therapies, which is rarely the case in clinical practice.^10^

**Figure 1.**
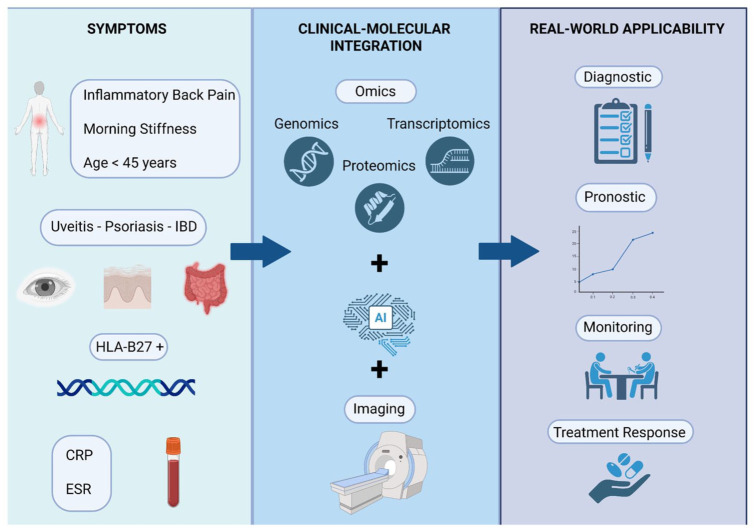
Schematic representation of the integration of clinical symptoms, molecular profiling, and imaging approaches for real-world applicability in patients with suspected spondyloarthritis. Clinical features (inflammatory back pain, morning stiffness, age <45 years, uveitis, psoriasis, inflammatory bowel disease, HLA-B27 positivity, elevated CRP/ESR) are combined with multi-omics analyses (genomics, transcriptomics, proteomics), artificial intelligence-based integration, and imaging. This comprehensive strategy enables improved diagnostic accuracy, prognostic assessment, disease monitoring, and evaluation of treatment response. CRP, C-reactive protein; HLA-B27, human leukocyte antigen-B27.

The goal of precision medicine is to improve clinical outcomes for individual patients and minimize unnecessary side effects for those less likely to respond to a particular treatment.^9^ The availability of novel drugs with innovative mechanisms of action and therapeutic strategies highlights the importance of early identification of patients who may respond better to one drug over another.^11^ The integration of omics technologies, advanced imaging techniques, and AI is expected to enhance diagnostic accuracy, prognostic assessment, patient monitoring, and personalized therapy, thereby bringing us closer to precision medicine in axSpA (Figure 1).

For many oncological conditions, patient-specific targeted therapies have brought significant clinical benefits. However, despite the use of advanced technologies, similar success has not yet been achieved for rheumatic diseases.^12^ Precision medicine for rheumatic diseases is challenging due to their polygenic nature and the complex interaction of genetic and environmental factors. Nonetheless, personalized medicine is increasing its interest in the field of Rheumatology,^13^ and some of these aspects are already used to guide treatment and monitor disease activity, such as, for example, autoantibodies are often used to aid diagnosis and for prognosis in autoimmune diseases.^14^

Rheumatic diseases are traditionally diagnosed through clinical assessment and standard laboratory markers. However, their heterogeneity often challenges accurate classification. In axSpA, conventional biomarkers like CRP, ESR, and HLA-B27 have diagnostic and prognostic limitations. Omics technologies offer a promising solution, enabling more precise biomarker discovery. Integrating genomics, epigenomics, transcriptomics, and proteomics may improve diagnosis, prognosis, and clinical decision-making in axSpA.^15–17^

## Imaging in axSpA: From diagnosis to disease staging

According to current recommendations, conventional sacroiliac joint radiography remains the first imaging test to perform in a patient with suspected axSpA.^18^ The limitation that this entails is that radiography is only capable of detecting irreversible and advanced structural damage, and the interpretation of the image is observer-dependent.^19^

To avoid delay in diagnosis, the use of magnetic resonance imaging (MRI) as a tool in the assessment of the sacroiliac joint was proposed since it detects both active and structural lesions.^20^ However, the problem of the interpretation of MRI arises, which is not always simple and sometimes leads to overdiagnosis. The presence of bone edema has been observed in healthy individuals, postpartum women, and runners, among other pathologies that mimic the findings of a positive MRI.^21^ In line with recent data-driven analyses, MRI can be prioritized as first-line in younger patients with shorter symptom duration, whereas radiography may be reasonable in older patients with longer duration—reflecting evidence-based cutoffs for modality choice.^22^ Beyond MRI, other imaging techniques such as computed tomography (CT) and positron emission tomography (PET) have shown potential for improving axSpA diagnosis. Low-dose CT (LDCT) has emerged as a valuable imaging modality for assessing structural damage in axSpA. Unlike conventional radiography, which is limited to detecting structural features in the anterior vertebral corners of the cervical and lumbar vertebrae, LDCT provides a more detailed and comprehensive evaluation of syndesmophytes and ankylosis along the entire vertebral column, including the thoracic spine, which is often obscured in standard X-rays.^23^ Despite its advantages, concerns remain regarding radiation exposure, although LDCT protocols have reduced doses to approximately 4 mSv—considerably lower than conventional CT scans but still higher than standard radiography.^24^ In addition, PET combined with CT (PET/CT) has emerged as a promising tool for detecting inflammatory activity by targeting metabolic changes associated with inflammation. Recent articles underscore the adjunctive role of PET/CT in axSpA, including studies embedding PET-CT within interventional designs.^25^ Studies using radiolabeled tracers, such as fluorodeoxyglucose, have demonstrated increased uptake in active SpA lesions, suggesting a potential role in differentiating inflammatory from non-inflammatory processes.^26^ Furthermore, novel imaging modalities like susceptibility-weighted imaging in MRI and synthetic CT (“bone MRI”) are being investigated to enhance the detection of structural damage in the sacroiliac joints and spine.^17^

In routine clinical practice, modality selection should be guided by the clinical question. When early inflammatory assessment is required, or in patients with a high pre-test probability of axSpA, MRI of the sacroiliac joints is the appropriate first-line examination.^22^ When the objective is to characterize structural progression or when MRI is contraindicated or inconclusive, LDCT can be used selectively to provide high-resolution depiction of bony changes^23^; because LDCT does not visualize active inflammation, it should not replace MRI for early inflammatory diagnosis or for monitoring disease activity. In situations of MRI–clinical discordance, or when metabolic characterization is pertinent, PET/CT may be used as an adjunct rather than as a first-line test, taking into account cost, availability, and tracer specificity.^25^ Finally, emerging AI/radiomics tools can contribute as clinician-in-the-loop second readers to improve consistency and triage; at present, they require external multicenter validation, calibration, and demonstration of clinical utility before routine deployment.^27^

Beyond its diagnostic value, imaging also plays a crucial role in disease staging in axSpA, particularly in identifying different levels of inflammatory activity. MRI has been shown to effectively differentiate between active and inactive axSpA. A study by Xin et al.^28^ developed a radiomics model using fat-suppressed T2-weighted MRI of the sacroiliac joints, which accurately distinguished active from non-active axSpA with high accuracy, sensitivity, and specificity. This suggests that MRI-based radiomics can be a valuable tool in staging disease activity.^28^ Diffusion-weighted imaging (DWI) and diffusion kurtosis imaging (DKI) have also been utilized to evaluate disease activity in axSpA. Xie et al.^28^ demonstrated that histogram parameters such as mean diffusivity and mean kurtosis could differentiate active from inactive disease and correlated with clinical activity indices like BASDAI and hsCRP. This indicates that DWI and DKI can serve as imaging biomarkers for disease activity.^29^ Furthermore, MRI relaxometry combined with peripheral blood mucosal-associated invariant T (MAIT) cells has been explored for evaluating inflammatory activity in axSpA. Yang et al.^30^ found that the combined-parameter model of T1 mapping and %CD69 + MAIT cells provided high diagnostic efficacy in distinguishing between active and inactive disease.

**Figure 2.**
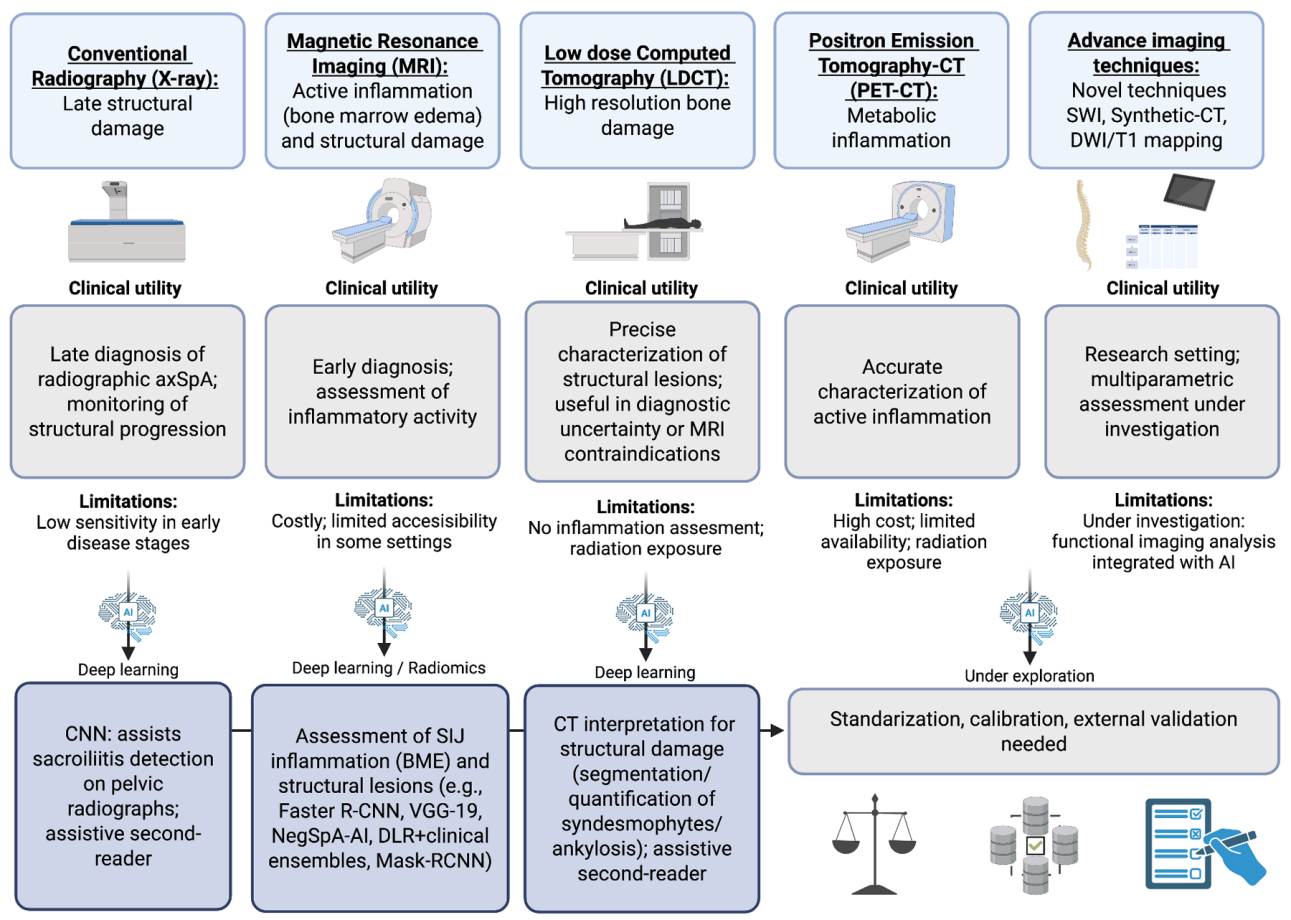
Overview of imaging techniques used in the assessment of axSpA. Conventional radiography detects late structural changes with low sensitivity. MRI identifies both active inflammation and structural lesions, enabling early diagnosis. Low-dose CT offers high-resolution visualization of bone damage but lacks sensitivity to inflammation. PET-CT allows for precise characterization of metabolic inflammation. Advanced imaging techniques such as SWI, synthetic CT, and DWI/T1 mapping are currently under investigation. AI tools, including CNN, radiomics, and predictive models (e.g., NegSpA-AI), are being developed to enhance the detection, classification, and integration of imaging and clinical data. AI, artificial intelligence; axSpA, axial spondyloarthritis; CNN, convolutional neural networks; CT, computed tomography; DWI, diffusion-weighted imaging; MRI, magnetic resonance imaging; PET, positron emission tomography; SWI, susceptibility-weighted imaging.

These studies highlight the potential of advanced imaging techniques to identify different levels of inflammatory activity in axSpA (Figure 2), which can be integrated with omics approaches for a comprehensive assessment of disease activity.

## The role of AI in imaging interpretation and precision medicine

AI might aid in the interpretation of the currently available imaging techniques. Several research groups are actively working in this field, and preliminary results suggest that AI-driven models could improve diagnostic accuracy by addressing challenges such as inter-reader variability and the differentiation of inflammatory and non-inflammatory changes. Most recently, Adam et al.^31^ explored the role of AI and machine learning in the diagnosis and management of axSpA, highlighting advancements in medical imaging, predictive modeling, and patient monitoring. Deep learning (DL), in particular, has demonstrated significant potential in enhancing diagnostic accuracy by assisting in the interpretation of X-rays, CT scans, and MRIs. Remarkably, some AI models have performed on par with or even surpassed radiologists in detecting sacroiliitis and other disease markers.^31^ Recent multicenter work reports AUCs approaching 0.96 for ankylosing spondylitis on pelvic radiographs—including robustness to smartphone-captured images—matching or outperforming expert readers, and systematic reviews confirm high performance across MRI/CT/X-ray while underscoring the need for external validation.^32,33^ Previously, Poddubnyy et al.^34^ investigated the development and diagnostic performance of a deep convolutional neural network (CNN) designed to detect radiographic sacroiliitis with expert-level accuracy. Evaluated in the OptiRef cohort, the study included 340 patients with chronic back pain, ultimately diagnosed with radiographic axSpA, non-radiographic axSpA, or non-SpA conditions. The CNN achieved a sensitivity of 79% and a high specificity of 94% in detecting radiographic sacroiliitis, demonstrating an 85% agreement with expert evaluation.^34^ In this context, CNNs have been employed to enhance MRI assessment, substantially improving diagnostic sensitivity and specificity. Advanced techniques, such as Faster R-CNN for region localization, VGG-19 for sacroiliitis classification, and maximum intensity projection for expert-level interpretation, further optimize performance. These advancements highlight AI’s potential to standardize MRI evaluation in accordance with ASAS criteria,^35^ facilitating early diagnosis and enabling more personalized treatment strategies for axSpA patients.^36^ AI-driven approaches enhance precision medicine by capturing complex variable interactions, enabling the development of predictive models for more effective patient selection in biologic therapy.^37^

In addition, the AI-driven model NegSpA-AI was developed to bridge the diagnostic gap in HLA-B27-negative axSpA patients, a subgroup frequently overlooked due to the absence of this genetic marker. By leveraging DL algorithms trained on sacroiliac joint MRI and clinical features, NegSpA-AI demonstrated superior diagnostic accuracy compared to junior clinicians, significantly improving their ability to distinguish axSpA from non-axSpA. The model not only outperformed independent radiologists and rheumatologists but also enhanced their diagnostic confidence and consistency when used as an assistive tool.^38^ Another recent study evaluated a deep learning radiomics (DLR) model that integrates multimodal MRI features and clinical data for diagnosing axSpA-related sacroiliitis. Using a dataset of 485 patients, CNN models (ResNet50, ResNet101, and DenseNet121) were trained and combined into an ensemble model, with ResNet50 demonstrating the best individual performance (AUC: 0.839, accuracy: 0.804). The final combined model, which incorporated the DLR signature with clinical factors, achieved superior diagnostic accuracy (AUC: 0.910, accuracy: 0.856) and strong clinical utility.^39^ Similarly, a DL model based on a Mask-RCNN architecture was trained using MRI data from the French DESIR cohort to detect bone marrow edema and predict active sacroiliitis according to the ASAS MRI criteria. The model achieved expert-level performance, particularly at baseline, with AUC values up to 0.98 and strong agreement with expert consensus (Matthews correlation coefficient: 0.90), underscoring the potential of AI to support imaging-based diagnosis in axSpA.^35^ Furthermore, the performance of a previously trained algorithm was validated in a large external cohort of 731 patients from the RAPID-axSpA and C-OPTIMISE trials, including both radiographic and non-radiographic axSpA. The model showed acceptable diagnostic capacity compared to central expert readings (sensitivity: 70%, specificity: 81%, Cohen’s kappa: 0.49), which supports feasibility while underscoring the need for further standardization and calibration.^40^ Beyond diagnosis, machine-learning models combining clinical, imaging, and multi-omics features report showed promising results for predicting response to biologics/JAK inhibitors and for risk stratification/triage; these remain assistive and require multicenter external validation and impact evaluation before routine use.^41,42^

### Validation, bias, and readiness for clinical use

Despite promising results, most AI studies to date are retrospective and single-center, which limits generalizability.^31^ Common risks include spectrum bias, class imbalance, and data leakage between training and testing.^43^ Robust development and evaluation should follow prespecified pipelines, include multi-center external validation, and report both discrimination (e.g., AUC, sensitivity/specificity) and calibration (e.g., calibration curves, calibration-in-the-large).^44^ Beyond static performance, teams should assess dataset drift and implement monitoring for performance degradation over time.^45^ Clinical utility should be demonstrated with decision-curve analysis and, ideally, prospective impact studies showing improvements in time-to-diagnosis, treatment selection, or resource use.^46^ Given these considerations and consistent with current evidence, including large external evaluations^40^ AI is best positioned as an assistive second-reader with clinician-in-the-loop, rather than a stand-alone diagnostic system; local governance and reporting standards in medical imaging are recommended prior to routine deployment.^47,48^

Advanced imaging in axSpA provides a direct visualization of inflammation and structural damage, enabling the identification of distinct disease patterns. However, integrating molecular data is essential to understanding the mechanisms driving these radiologic changes. Omics technologies offer insights into genetic, epigenetic, and expression-level alterations that underlie inflammation and bone remodeling. Correlating imaging findings with molecular profiles enhances our understanding of disease pathogenesis and supports the development of personalized medicine strategies, improving diagnosis, prognosis, and treatment decisions.

## Integrating imaging and omics: Toward a unified precision medicine framework in AxSpA

While imaging modalities and omics technologies have independently advanced the understanding of axSpA, their integration into a unified precision medicine framework remains largely underexplored. Increasing evidence suggests that combining imaging data with multi-omics profiles, including genomics, transcriptomics, proteomics, and metabolomics, could significantly enhance the identification of robust, disease-specific biomarkers for diagnosis, stratification, and therapeutic guidance.

Several recent reviews and studies support the clinical potential of this integrative approach. Allard-Chamard et al.^49^ highlighted the utility of advanced molecular tools—such as polygenic risk scores (PRS), single-cell RNA sequencing, and spatial transcriptomics—in conjunction with imaging data to correlate molecular profiles with radiologic phenotypes, thereby enabling more precise disease characterization. Similarly, Minopoulou et al.^50^ emphasized the added value of high-resolution and functional imaging techniques, particularly PET, in offering both anatomical and immunometabolic assessments of inflammation. When coupled with omics data, these modalities may allow for more accurate monitoring and earlier, personalized intervention in axSpA.^50^ Moreover, Brown et al.^16^ have pointed to the limitations of current biomarker panels in axSpA and proposed that integrating imaging with multi-omics data could provide more specific and clinically relevant signatures, facilitating better prediction of disease progression and response to treatment.^16^

Despite these promising insights, the clinical implementation of such integrative strategies remains limited. To bridge this gap, future research should prioritize the development of harmonized, multimodal datasets and advanced computational pipelines capable of integrating high-dimensional data. Establishing robust connections between radiological phenotypes and underlying molecular mechanisms may accelerate the transition from descriptive imaging-based evaluation to biologically informed clinical decision-making. Ultimately, this convergence represents a key step toward the realization of truly personalized medicine in axSpA.

In this context, multi-omics technologies emerge as a powerful tool to decode the molecular complexity of axSpA. By capturing complementary layers of biological information—ranging from genomic susceptibility to epigenetic regulation, transcriptional activity, protein function, and metabolic profiles—omics approaches offer an unprecedented opportunity to uncover disease mechanisms, identify predictive biomarkers, and refine patient stratification strategies.

## Genomics

Before the advent of genome-wide association studies, limited research existed on the association of specific genetic backgrounds with this disease, emphasizing from the outset the well-known association with the *HLA-B27* gene in axSpA disease pathogenesis. A study by Rubin et al.,^51^ firmly established a significant linkage between the major histocompatibility complex (MHC) region and axSpA, thereby underlining the direct involvement of the *HLA-B27* gene in the disease’s pathogenesis. Subsequent studies have suggested that certain genetic polymorphisms in matrix metalloproteinase-3 and TIMP metallopeptidase inhibitor 1 may influence the development of axSpA,^52^ while others have explored how endoplasmic reticulum aminopeptidase 1 (*ERAP-1*) gene polymorphisms affect gene expression levels in antigen-presenting cells.^53^

In 2010, the Australo-Anglo-American SpA Consortium identified four genetic loci associated with axSpA risk, emphasizing the role of IL-23 and IL-1 cytokine pathways. The study, involving over 2000 axSpA patients and 5000 controls, was validated in an independent cohort, confirming the involvement of interleukin-23 receptor (*IL23R*) and *ERAP1* in disease pathogenesis.^54^ Evans et al.^55^ identified strong associations between axSpA and variants in the RUNX family transcription factor 3 (*RUNX3*), lymphotoxin beta receptor-tumor necrosis factor receptor superfamily member 1A (*LTBR-TNFRSF1A*), and interleukin 12 subunit beta (*IL12B*) regions, as well as significant associations at prostaglandin E receptor 4 (*PTGER4*), TBK1-binding protein 1 (*TBKBP1*), anthrax toxin receptor 2 (*ANTXR2*), and caspase recruitment domain family member 9 (*CARD9*) loci. In addition, ERAP1 polymorphisms were found to influence axSpA risk specifically in HLA-B27-positive individuals.^55^ New susceptibility loci near EDIL3-HAPLN1 and within anoctamin 6 (ANO6) were identified in the Chinese population, implicating genes involved in bone and cartilage development. Previously reported MHC associations in Europeans were also confirmed.^56^ A study of 10,619 axSpA cases and 15,145 controls identified 13 new risk loci and 12 haplotypes across 11 loci, including two regions encoding aminopeptidases involved in MHC class I presentation; protective variants reduced enzyme activity and increased MHC I surface expression.^57^ Ellinghaus et al.^58^ conducted the largest cross-disease genetic study on chronic immune-mediated diseases, identifying 17 new susceptibility loci for axSpA and 3 novel risk loci. Their findings expanded the known risk loci for axSpA to 48, revealing disease-specific patterns at shared genetic regions.^58^ Besides, Nam et al.^59^ analyzed SNPs in 444 axSpA patients and identified variants, including in RYR3 and ZC3H11A, linked to severe radiographic damage, highlighting the PI3K-Akt pathway in disease progression.

PRSs combine genetic variants to estimate individual susceptibility to complex diseases. While mainly used in research, they show potential for clinical use, particularly in early disease stages, by supporting diagnosis and treatment decisions.^60^ PRSs validated in European and East Asian populations outperformed traditional markers such as HLA-B27, MRI, and CRP in predicting axSpA, with AUCs of 0.924 in Europeans and 0.948 in East Asians. In Europeans, PRS achieved higher positive (78.2%) and negative (100%) predictive values compared to HLA-B27 (51.9% and 97.9%). These findings highlight the superior accuracy of PRS and its potential for early diagnosis, while also underscoring the importance of developing population-specific models for clinical implementation.^61^

Genome-wide association studies have identified multiple susceptibility loci, including but not limited to HLA-B27, refining the genetic basis of axSpA (Table 1). However, genetic predisposition alone does not fully explain disease onset. Epigenetic mechanisms, such as DNA methylation, regulate gene activity and mediate gene–environment interactions, bridging genetic susceptibility with disease expression.

**Table 1.** Genetic landscape in axSpA patients.

Functional group	Gene/nearby gene	SNP/allele	Locus	Study population	Association strength	Citation
Antigen presentation and HLA-related	*ERAP1*	rs30187	5q15	1787 axSpA and 4800 HCs (discovery cohort); 3023 axSpA and 8779 HCs (replication cohort)	1.8 × 10^−27^	39, 41
	*FCGR2A*	rs1801274	1q23	100,619 axSpA and 15,145 HCs	1.4 × 10^−9^	43
	*UBE2E3*	rs12615545	2q31	100,619 axSpA and 15,145 HCs	1.0 × 10^−9^	43
	*HLA-B27*	HLA-B27 alleles	NA	97 individuals from 13 Caucasian axSpA families and 59 individuals from 2 Newfoundland families	NA	37
Bone formation and structural damage	*EDIL3-HAPLN1*	rs4552569	5q14.3	1837 axSpA and 4231 HCs (discovery cohort); 2100 axSpA and 3496 HCs (validation cohort)	8.77 × 10^−10^	42
	*ANTXR2*	rs4333130	4q21.21	2053 axSpA and 5140 HCs (discovery cohort); 898 axSpA and 1518 HCs (replication cohort)	9.3 × 10^−8^	40
	*RUNX3*	rs11249215	1p36	1787 axSpA and 4800 HCs (discovery cohort); 3023 axSpA and 8779 HCs (replication cohort)	9.2 × 10^−11^	41
	*PTGER4*	rs10440635	5p13	1787 axSpA and 4800 HCs (discovery cohort); 3023 axSpA and 8779 HCs (replication cohort)	2.6 × 10^−7^	41
	*ANO6*	rs17095830	12q12	1837 axSpA and 4231 HCs (discovery cohort); 2100 axSpA and 3496 HCs (validation cohort)	1.63 × 10^−8^	42
IL-23/Th17 and cytokine signaling	*SH2B3*	rs11065898	12q24	100,619 axSpA and 15,145 HCs	4.7 × 10^−8^	43
	*IL6R*	rs4129267	1q21	100,619 axSpA and 15,145 HCs	3.4 × 10^−13^	43
	*IL12B*	rs6556416	5q33	1787 axSpA and 4800 HCs (discovery cohort); 3023 axSpA and 8779 HCs (replication cohort)	1.9 × 10^−8^	41
	*IL23R*	rs11209026	1p31.3	2053 axSpA and 5140 HCs (discovery cohort); 898 axSpA and 1518 HCs (replication cohort)	9.1 × 10^−14^	40
	*TYK2*	rs35164067	19p13	100,619 axSpA and 15,145 HCs	3.4 × 10^−10^	43
Immune regulation and T-cell signaling	*ICOSLG*	rs7282490	21q22	100,619 axSpA and 15,145 HCs	6.2 × 10^−9^	43
	*BACH2*	rs17765610	6q15	100,619 axSpA and 15,145 HCs	5.3 × 10^−8^	43
	*LTBR-TNFRSF1A*	rs11616188	12p13	1787 axSpA and 4800 HCs (discovery cohort); 3023 axSpA and 8779 HCs (replication cohort)	4.1 × 10^−12^	41
	*ZMIZ1*	rs1250550	10q22	100,619 axSpA and 15,145 HCs	1.5 × 10^−9^	43
	*IL27-SULT1A1*	imm_16_28525386	16p11	100,619 axSpA and 15,145 HCs	2.6 × 10^−9^	43
Inflammation and innate immunity	*IL1R2*	rs2310173	2q11.2	2053 axSpA and 5140 HCs (discovery cohort); 898 axSpA and 1518 HCs (replication cohort)	4.8 × 10^−7^	40
	*CARD9*	rs10781500	9q34	1787 axSpA and 4800 HCs (discovery cohort); 3023 axSpA and 8779 HCs (replication cohort)	1.1 × 10^−6^	41
	*MMP-3*	6A/6A	NA	241 axSpA and 241 HCs	2.41-fold (95% CI 1.55–3.74)	38
	*TIMP-1*	C alleles	NA	241 axSpA and 241 HCs	OR = 1.28, 95% CI 0.92–1.77	38
	*TBKBP1*	rs8070463	17q21	1787 axSpA and 4800 HCs (discovery cohort); 3023 axSpA and 8779 HCs (replication cohort)	5.3 × 10^−8^	41
Mucosal immunity and gut-joint axis	*NOS2*	rs2531875	17q11	100,619 axSpA and 15,145 HCs	1.2 × 10^−10^	43
	*GPR35*	rs4676410	2q37	100,619 axSpA and 15,145 HCs	9.9 × 10^−9^	43
	*GPR65*	rs11624293	14q31	100,619 axSpA and 15145 HCs	1.5 × 10^−10^	43
	*NKX2-3*	rs11190133	10q24	100,619 axSpA and 15,145 HCs	4.9 × 10^−14^	43

ANO6, anoctamin 6; ANTXR2, anthrax toxin receptor 2; axSpA, axial spondyloarthritis; BACH2, BTB domain and CNC homolog 2; CARD9, caspase recruitment domain family member 9; CI, confidence interval; EDIL3-HAPLN1, EGF-like repeats and discoidin domains 3-hyaluronan and proteoglycan link protein 1; ERAP1, endoplasmic reticulum aminopeptidase 1; FCGR2A, Fc gamma receptor IIa; GPR35, G protein-coupled receptor 35; GPR65, G protein-coupled receptor 65; HCs, healthy controls; HLA-B27, human leukocyte antigen-B27; ICOSLG, inducible T-cell costimulator ligand; IL12B, interleukin 12 subunit beta; IL1R2, interleukin 1 receptor type 2; IL23R, interleukin-23 receptor; IL27-SULT1A1, interleukin 17A-sulfotransferase family 1A member 1; IL6R, interleukin 6 receptor; LTBR-TNFRSF1A, lymphotoxin beta receptor-TNF receptor superfamily member 1A; MMP-3, matrix metalloproteinase-3; NA, not applicable; NKX2-3, NK2 homeobox 3; NOS2, nitric oxide synthase 2; OR, odds ratio; PTGER4, prostaglandin E receptor 4; RUNX3, RUNX family transcription factor 3; SH2B3, SH2B adaptor protein 3; TBKBP1, TBK1-binding protein 1; TIMP-1, TIMP metallopeptidase inhibitor 1; TYK2, tyrosine kinase 2; UBE2E3, ubiquitin conjugating enzyme E2 E3; ZMIZ1, zinc finger MIZ-type containing 1.

## Epigenomics

The epigenome regulates gene expression through chemical modifications and chromatin structure, influencing activation across cell types. Sequencing studies have linked epigenomic mutations to cancer and developmental disorders. Advances such as high-throughput methylome analysis and machine learning are improving insights into disease mechanisms and biomarker discovery.^62^ Epigenetic research in SpA remains limited, with most studies focusing on known genetic risk factors and involving small sample sizes that need further validation. Existing findings mainly come from analyses of peripheral blood mononuclear cells (PBMCs), whole blood, or serum.^63^

In PBMCs, several genes have been identified as differentially methylated in patients with axSpA. DNA methyltransferase 1 (DNMT1) showed increased promoter methylation in axSpA patients, inversely correlating with its expression.^64^ Similarly, the *BCL11B* gene exhibits increased methylation and reduced expression in axSpA patients.^65^ Zhang et al.^66^ found IL12B hypermethylation at two CpG sites in axSpA patients, associated with HLA-B27 positivity and male sex, though with limited specificity and sensitivity for axSpA diagnosis. Furthermore, a genome-wide methylation analysis of PBMCs from five axSpA patients and five controls revealed that the *HLA-DQB1* gene showed the most significant differential methylation signal (cg14323910, adjusted *p* = 1.84 × 10⁻⁶, β difference = 0.5634) associated with axSpA.^67^ In a more extensive study involving 45 individuals and a validation cohort of 24 individuals, researchers identified and validated 2526 differentially methylated positions (DMPs) in axSpA patients compared to controls, with a notable enrichment in genes related to T-cell receptor signaling and Th17 differentiation pathways. In addition, 158 DMPs correlated with mRNA expression, suggesting potential molecular targets for axSpA.^68^

In whole blood, interferon regulatory factor 8 showed promoter hypermethylation and reduced expression in axSpA compared to controls, correlating with disease duration, BASFI, and ESR.^69^ As expected, some researchers have also focused on the DNA methylation and expression of the *ERAP1* gene. In a study involving 100 axSpA patients and 100 healthy controls (HCs), two CpG sites within ERAP1 were significantly hypermethylated in axSpA patients, corresponding with significantly reduced gene expression. The highest AUC found for diagnosing axSpA patients was 0.779.^70^ In addition, other genes have been identified with altered methylation patterns in axSpA. For example, hypermethylation has been observed in CpG islands of the *TRAF5*^71^ and *PDCD1*^72^ genes, while hypomethylation has been noted in genes such as Forkhead box O3 (*FOXO3a*)^73^ and RUNX Family Transcription Factor 2 (*RUNX2*).^74^

Interestingly, a genome-wide DNA methylation analysis of 24 axSpA individuals (12 HLA-B27+, 12 HLA-B27−) and 12 osteoarthritis controls identified 67 differentially methylated sites between axSpA and controls. Hypermethylated genes were linked to GTPase activity, while hypomethylated genes included HCP5. Compared to HLA-B27− axSpA patients, HLA-B27+ cases showed strong hypomethylation in HLA complex P5 (HCP5), tubulin folding cofactor A (TBCA), and phospholipase D family member 6 (PLD6). The authors suggested that HLA-B27 may contribute to axSpA by inducing epigenetic dysregulation.^75^ The complete epigenomic landscape observed in axSpA patients is summarized in Table 2.

**Table 2.** DNA methylation alterations in genes associated with axSpA.

Gene	Population size	Biological Sample	Methylation status	mRNA expression change	Citation
*DNMT1*	40 axSpA and 40 HCs	PBMCs	Differentially methylated	Downregulated	64
*BCL11B*	50 axSpA and 50 HCs	PBMCs	Hypermethylated	Downregulated	65
*IL12B*	99 axSpA and 99 HCs	PBMCs	Hypermethylated	Upregulated	66
*HLA-DQB1*	5 axSpA and 5 HCs	PBMCs	Differentially methylated	NA	67
*IRF8*	99 axSpA and 99 HCs	Whole blood	Hypermethylated	Downregulated	69
*ERAP1*	100 axSpA and 100 HCs	Whole blood	Hypermethylated	Downregulated	70
*TRAF5*	98 axSpA and 98 HCs	Whole blood	Hypermethylated	Downregulated	71
*PDCD1*	80 axSpA and 80 HCs	Whole blood	Hypermethylated	Downregulated	72
*FOXO3a*	84 axSpA and 83 HCs	Whole blood	Hypomethylated	Downregulated	73
*RUNX2*	83 axSpA and 83 HCs	Whole blood	Hypomethylated	Upregulated	74
*HCP5*	24 axSpA and 12 OA	Whole blood	Hypomethylated	NA	75
*TBCA*		Whole blood	Hypomethylated		
*PLD6*		Whole blood	Hypomethylated		

axSpA, axial spondyloarthritis; BCL11B, B-cell lymphoma/leukemia 11B; DNMT1, DNA methyltransferase 1; ERAP1, endoplasmic reticulum aminopeptidase 1; FOXO3a, Forkhead box O3; HCs, healthy controls; HCP5, HLA complex P5; HLA-DQB1, major histocompatibility complex, class II, DQ beta 1; IL12B, subunit beta of interleukin 12; IRF8, interferon regulatory factor 8; LGR6, leucine rich repeat containing G protein-coupled receptor 6; NA, not applicable; OA, osteoarthritis; PBMCs, peripheral blood mononuclear cells; PDCD1, programmed cell death protein 1; PLD6, phospholipase D family member 6; RUNX2, RUNX family transcription factor 2; SOCS1, suppressor of cytokine signaling 1; TBCA, tubulin folding cofactor A; TRAF5, TNF receptor associated factor 5.

## Transcriptomics

The transcriptome reflects cellular identity through gene expression patterns and regulatory networks. While technologies like single-cell RNA sequencing and spatial transcriptomics have improved understanding of SpA, the transcriptomic landscape of the disease remains underexplored, underscoring the need for further research.

Transcriptomic studies have revealed molecular mechanisms in axSpA, identifying 973 differentially expressed genes (DEGs) in PBMCs enriched in immune and inflammatory pathways, especially TNFα and NF-κB signaling. IL-6 emerged as a potential diagnostic biomarker, emphasizing the role of immune dysregulation in the disease.^76^ Similarly, Li et al. focused on lncRNAs and mRNAs in PBMCs, detecting 114 upregulated and 45 downregulated lncRNAs, alongside 284 upregulated and 435 downregulated mRNAs. Their findings highlighted enrichment in B-cell receptor, TNF, and NF-κB signaling pathways, emphasizing the interplay between lncRNAs and immune regulation in axSpA.^77^ Tissue-specific analysis revealed upregulation of Piezo1 in axSpA spinal ligaments, suggesting a role in mechanical stress response.^78^ Similarly, Li et al.^79^ found enrichment of extracellular matrix-related pathways, such as extracellular matrix organization.

Single-cell technologies like single-cell RNA sequencing (scRNA-seq) and single-cell assay for transposase-accessible chromatin sequencing (scATAC-seq) have uncovered 18 distinct PBMC cell types in axSpA, offering detailed insight into cellular heterogeneity and gene regulation. These analyses identified NF-κB, primarily derived from CD8+ T cells, as a central mediator of dysregulated TNF signaling. In addition, altered binding sites for transcription factors Fos proto-oncogene (FOS), Jun proto-oncogene (JUN), and JunB proto-oncogene (JUNB) pointed to disruptions in the TNF-NF-κB pathway, a key hallmark of axSpA pathogenesis.^80^

Focusing on spinal entheses, Feng et al.^81^ found that transcription factors C-JUN, C-FOS, and caveolae-associated protein 1 (CAVIN1) are upregulated in axSpA and promote osteogenesis in mesenchymal stem cells, potentially driving pathological bone formation. They also identified a novel early-stage neutrophil subpopulation (CD99_G1) elevated in axSpA, revealing a new inflammatory component of the disease.^81^ In the context of natural killer (NK) cells, a distinct immune imbalance has been identified in axSpA patients, characterized by a reduction in total NK cells and the CD56dim subset, while the CD56bright subset was increased. This shift in NK cell populations suggests a potential role in immune dysregulation and chronic inflammation in axSpA.^82^ Moreover, Tang et al.^83^ demonstrated that killer cell lectin-like receptor C2 (NKG2C+) CD8+ T cells, an aging-associated subset, exhibit enhanced cytotoxicity in ankylosing spondylitis, driven by HLA-B27-induced activation of the PI3K-Akt pathway. Their findings link immune aging with AS pathogenesis and suggest that NKG2C blockade could represent a novel therapeutic strategy.^83^ Finally, a recent advance by Liu et al. using single-cell RNA sequencing and spatial transcriptomics of synovial tissue from axSpA and psoriatic arthritis patients identified CD4+ tissue-resident memory Th17 (TRM17) cells as the predominant source of IL-17A. They further showed interactions with CLEC10A+ dendritic cells and IL-17A-driven fibroblast activation, reinforcing the central role of the IL-17 pathway and pointing to TRM17 cells and their epigenetic regulation (via BRD1) as potential therapeutic targets for sustained disease remission.^84^

Transcriptomic studies, particularly those using single-cell technologies, have significantly advanced our understanding of axSpA by identifying key DEGs, signaling pathways, and previously unrecognized cell populations involved in disease pathogenesis. The key studies on high-throughput transcriptomic analysis in axSpA are summarized in Table 3.

**Table 3.** Transcriptomic advanced studies in axSpA.

Altered transcriptome	Study population	Biological sample	Key points	Methodology	Citation
973 DEGs (644 upregulated, 329 downregulated)	5 axSpA and 5 HCs	PBMCs	DEGs enriched in immune and inflammatory pathways, notably TNF and NF-κB signaling	RNA-seq	76
114 upregulated and 45 downregulated lncRNAs; 284 upregulated and 435 downregulated mRNAs	6 axSpA and 6 HCs	PBMCs	Enriched in DNA binding, protein binding, immune response, and inflammatory response	RNA-seq	77
Piezo1	14 axSpA and 14 Cs	Spinal ligament tissue	Piezo1-mediated mechanotransduction promotes entheseal pathological new bone formation	RNA-seq	78
TNC	10 axSpA and 12 HCs	Spinal ligament tissue	TNC promoted new bone formation by enhancing chondrogenic differentiation during endochondral ossification	RNA-seq	79
NF-kB, FOS, JUN, and JUNB	6 axSpA and 6 HCs	PBMCs	Identified 18 distinct PBMC cell types. Possible mechanism by which NF-kB abnormally regulates FOS, JUN, and JUNB and drives axSpA progression	scRNA-seq and scATAC-seq	80
C-JUN, C-FOS, and CAVIN1	5 axSpA and 3 HCs	Spinal enthesis	Associated with osteogenesis in axSpA. A novel subcluster of early-stage neutrophils, CD99_G1, was elevated in axSpA	scRNA-seq	81
Impaired cytotoxic genes in NK cells	29 axSpA and 29 HCs	PBMCs	Reduction in total NK cells and CD56dim NK subset	scRNA-seq	82
PI3K-Akt pathway	87 AxSpA	PBMCs	Activation of the PI3K-Akt pathway in NKG2C + CD8 + T cells by HLA-B27	scRNA-seq	83
IL-17A	5 AxSpA and 6 PsA	Synovial tissue	CD4 + CXCR6+ TRM17 cells are the predominant spontaneous IL17A producers in SpA synovium	scRNA-seq	84

Akt, protein kinase B; axSpA, axial spondyloarthritis; CAVIN1, caveolae-associated protein 1; CD, cluster differentiation; C-FOS, C-Fos proto-oncogene; C-JUN, C-Jun proto-oncogene; CXCR6, C-X-C motif chemokine receptor 6; DEGs, differentially expressed genes; FOS, Fos proto-oncogene; HCs, healthy controls; HLA-B27, human leukocyte antigen B27; IL17A, interleukin 17A; JUN, Jun proto-oncogene; JUNB, JunB proto-oncogene; lncRNAs, long non-coding RNAs; NF-kB, nuclear factor kappa B; NK, natural killer cells; NKG2C, killer cell lectin-like receptor C2; PBMCs, peripheral blood mononuclear cells; PI3K, phosphatidylinositol-4,5-bisphosphate 3-kinase; PsA, psoriatic arthritis; scATAC-seq, single-cell assay for transposase-accessible chromatin sequencing; scRNA-seq, single-cell RNA sequencing; TNC, tenascin-C; TNF, tumor necrosis factor; TRM17, tissue-resident memory Th17.

## Proteomics

The proteome, encompassing the complete set of proteins expressed by the genome at a given time, is fundamental to cellular function and identity. Unlike genes, proteins are the active molecules that drive biological processes, and their abundance, modifications, and interactions provide a direct reflection of cellular states and regulatory mechanisms.^85^ Proteomic analyses reveal how cells respond to stimuli and undergo differentiation, shedding light on disease-related disruptions. Advances in large-scale and single-cell proteomics, particularly mass spectrometry-based methods, have greatly improved the resolution of protein expression profiling.^86^ The key studies utilizing high-throughput proteomic techniques are summarized in Table 4.

**Table 4.** Key studies utilizing advanced proteomic techniques in axSpA.

Protein	Study population	Biological sample	Key points	Methodology	Citation
TNF and FKBPL	2860 axSpA and 270,964 HCs	Plasma	AxSpA susceptibility/potential therapeutic target	Proteome-wide MR study/GWAS	87
MAPK14	2860 axSpA and 270,964 HCs	Plasma	AxSpA susceptibility/potential therapeutic target	Proteome-wide MR study/GWAS	88
IL7R and IL23R	19,688 axSpA and 15,145 HCs	Plasma	AxSpA susceptibility/potential therapeutic target	Proteome-wide MR study/GWAS	89
CD63 and CD81	40 axSpA and 28 HCs	Plasma exosomes	CD63 and CD81 markers were elevated in axSpA with respect to controls. Exosomes from patients with axSpA inhibited the proliferation of Treg	Western blotting and cytokine assay	90
SAA1	30 axSpA and 30 HCs	Serum extracellular vesicles	Potential diagnostic biomarker (AUC of 0.768)	LC-MS/MS and ELISA	91
IGHD, RBM8A, AHSG, SEC24C, and FGG (top 5)	9 gout, 9 RA, 9 axSpA, and 9 OA	SF exosomes	84 differentially expressed proteins in SF-derived exosomes are significantly involved in the citrate cycle	LC-MS/MS and ELISA	92
Haptoglobin	32 RA and 24 axSpA	SF	Higher levels of SpA SF than RA SF	LC-MS/MS	93
HSP90 and HLA-E	9 axSpA and 9 HCs	PBMCs	89 differentially expressed and phosphorylated proteins enriched in antigen processing pathways	LC-MS/MS	94
HSP90AB1, HSP90AA1, HSPA8, ITPR1, MYLK, STIM1, MYL12A, MYL9, and ROCK2	9 axSpA and 9 HCs	PBMCs	Key proteins involved in antigen processing and presentation, platelet activation, and leukocyte transendothelial migration	LC-MS/MS	95
CTSG, DEFA3, PTPRC, and PRDX1	12 axSpA and 9 HCs	PBMCs	Proteins involved in the biological process of cell killing	iTRAQ, LC-MS/MS, and ELISA	96
MYD88, DEFA3, TLR2, and RELA subunit of NF-kB	34 axSpA (20 treatment naïve + 14 treated with NSAIDs and DMARDs) and 19 HCs	PBMCs	Altered glycolytic enzymes, lipid transporters, and chemokine signaling	Shot-gun proteomics and LC-MS/MS	97
MPO	6 axSpA and 6 controls	Hip ligament	MPO overexpression may drive hip joint inflammation in axSpA via the phagosome pathway	LC-MS/MS	98
CRP, haptoglobin, and apolipoproteins (APOD, APOA2, and APOA1)	35 biologic-naïve axSpA and 14 pre/post-TNFi treatment	Plasma	CRP and haptoglobin levels decreased, while apolipoproteins increased in TNFi responders	LC-MS/MS	99
CRP and SAA1	Cohort 1: 15 axSpA and 60 HCs; Cohort 2: 138 active axSpA 190 HCs	Serum	The combination of CRP and SAA1 shows high potential for diagnosing disease status	TMT-based proteomics	100
CDCP1, IL6, and PON3	245 axSpA	Serum	IL-6, CDCP-1, and PON-3 emerge as key mediators connecting inflammation to CV risk.	PEA	101

AHSG, alpha-2-HS-glycoprotein (Fetuin-A); APO, apolipoprotein; axSpA, axial spondyloarthritis; CD, cluster of differentiation; CDCP1, CUB domain-containing protein 1; CRP, C-reactive protein; CTSG, cathepsin G; CV, cardiovascular; DEFA3, defensin alpha 3; DMARDs, disease-modifying antirheumatic drugs; ELISA, enzyme-linked immunosorbent assay; FGG, fibrinogen gamma chain; FKBPL, FK506-binding protein-like; GWAS, genome-wide association study; HC, healthy controls; HLA-E, human leukocyte antigen-E; HSP90, heat shock protein 90; HSP90AA1, heat shock protein 90 alpha family class A member 1; HSP90AB1, heat shock protein 90 alpha family class B member 1; HSPA8, heat shock protein family A (Hsp70) member 8; IGHD, immunoglobulin heavy constant delta; IL23R, interleukin 23 receptor; IL6, interleukin 6; IL7R, interleukin 7 receptor; ITPR1, inositol 1,4,5-trisphosphate receptor type 1; LC-MS, liquid chromatography-mass spectrometry; MAPK14, mitogen-activated protein kinase 14; MPO, myeloperoxidase; MR, Mendelian randomization; MYD88, myeloid differentiation primary response 88; MYL12A, myosin light chain 12A; MYL9, myosin light chain 9; MYLK, myosin light chain kinase; NSAIDs, nonsteroidal anti-inflammatory drugs; OA, osteoarthritis; PBMC, peripheral blood mononuclear cells; PEA, proximity extension assay; PON3, paraoxonase 3; PRDX1, peroxiredoxin 1; PTPRC, protein tyrosine phosphatase receptor type C (CD45); RBM8A, RNA-binding motif protein 8A; RELA, RELA proto-oncogene, NF-κB subunit; ROCK2, Rho-associated coiled-coil containing protein kinase 2; SAA1, serum amyloid A1; SEC24C, SEC24 homolog C, COPII-coated vesicle component; SF, synovial fluid; STIM1, stromal interaction molecule 1; TLR2, Toll-like receptor 2; TMT, tandem mass tag; TNF, tumor necrosis factor; TNFi, tumor necrosis factor inhibitor.

Several studies have shown that plasma proteins, extracellular vesicles (EVs), and immune cell proteomics play key roles in axSpA susceptibility and progression. Using approaches like Mendelian randomization (MR) and proteomic profiling, one large-scale study found that plasma proteins such as TNF and FK506-binding protein-like (FKBPL) were positively associated with axSpA risk, while AIF1 and ACOT13 showed negative associations. This analysis used data from over 54,000 participants in the UK Biobank. Similarly, a two-sample MR identified 1654 axSpA-related proteins.^87^ Similarly, a two-sample MR analysis identified 1654 axSpA-associated plasma proteins, highlighting mitogen-activated protein kinase 14 (MAPK14) as particularly significant, with colocalization analysis reinforcing MAPK14’s potential as a therapeutic target.^88^ Expanding on these findings, an integrative MR and protein–protein interaction analysis across multiple genome-wide association study (GWAS) datasets identified eight proteins, including IL7R and IL23R, that colocalized with axSpA genetic risk loci, suggesting novel therapeutic targets.^89^

Beyond plasma protein analyses, EVs and exosomes have emerged as key players in axSpA pathogenesis. Tavasolian et al.^90^ identified elevated CD63 and CD81 in axSpA-derived plasma exosomes, along with a distinct cytokine profile characterized by reduced IL-8 and IL-10. Supporting this, Huang et al.^91^ analyzed serum-derived EV proteins using liquid chromatography-mass spectrometry (LC-MS)/MS and ELISA, detecting 73 differentially expressed proteins (DEP). Notably, serum amyloid A1 (SAA1) was significantly elevated in axSpA-derived EVs, demonstrating a diagnostic potential (AUC = 0.768).^91^

The significance of exosomes extends beyond plasma, as their role in SF has also been explored. Huang et al. conducted a proteomic analysis of SF-derived exosomes in axSpA, identifying 84 DEPs. Among them, RNA-binding protein 8A and Sec24C were uniquely elevated and linked to the acute-phase response and citrate cycle.^92^ This aligns with findings by Birkelund et al.,^93^ who analyzed the SF proteome in SpA and RA. Their results showed that while SpA SF contained fewer inflammatory proteins than RA SF, it exhibited higher haptoglobin levels. Furthermore, plasma CRP levels reflected acute-phase responses in both diseases, highlighting inflammatory differences between SpA and RA.^93^

Immune cell proteomics has also advanced understanding of axSpA pathogenesis. Proteomic analysis of PBMCs from axSpA patients revealed immune dysregulation, with Lu et al.^94^ identifying 89 differentially expressed and phosphorylated proteins involved in antigen presentation and immune modulation, highlighting key roles for heat shock protein 90 (HSP90) and human leukocyte antigen-E (HLA-E) in early disease. Expanding on this, Yu et al.^95^ confirmed that antigen processing pathways were enriched in axSpA, with elevated levels of heat shock protein 90 alpha family class B member 1 (HSP90AB1), heat shock protein 90 alpha family class A member 1 (HSP90AA1), and heat shock protein family A (Hsp70) member 8 (HSPA8), while platelet activation and leukocyte migration pathways were also dysregulated. Next, Cai et al.^96^ utilized an iTRAQ-based proteomic approach to analyze PBMCs from axSpA patients and HCs. Their analysis identified 183 DEPs associated with acute inflammatory responses, including cathepsin G (CTSG), defensin alpha 3 (DEFA3), protein tyrosine phosphatase receptor type C (PTPRC), and peroxiredoxin 1 (PRDX1), primarily linked to immune-mediated cell killing.^96^

Wang et al. revealed widespread immune dysregulation in axSpA using mass cytometry and proteomics, including altered metabolism and immune signaling in PBMCs. Single-cell analysis highlighted dysregulation across immune cell subsets and key monocyte-T-cell interactions. Importantly, they identified two axSpA subtypes—treatment-naïve and treated patients—with distinct immune profiles, shedding light on disease heterogeneity and treatment response.^97^ Tissue-level analysis by Yu et al. identified 193 DEPs in hip ligaments of axSpA patients, linked to lysosomal and phagosome pathways. Myeloperoxidase was highlighted as a key protein, with in vitro data suggesting its upregulation promotes inflammation in fibroblasts.^98^

Proteomics has also contributed to assessing treatment responses in axSpA. Sobral et al.^99^ identified reductions in CRP and haptoglobin levels in TNFi responders, while apolipoproteins (APOD, APOA2, and APOA1) increased, suggesting potential markers for monitoring treatment efficacy.^99^ In addition, Liu et al.^100^ used tandem mass tag-based proteomics to distinguish active and stable axSpA from HCs, confirming CRP and SAA1 as reliable biomarkers of active disease with high diagnostic accuracy.

Finally, a recent study provides an example of how proteomics can be linked with imaging in axSpA. In 74 patients, carotid ultrasound was used to detect atherosclerotic plaques, while serum proteomic profiling identified proteins associated with vascular pathology. Notably, IL-6 and CUB domain-containing protein 1 (CDCP-1) correlated with both endothelial dysfunction and the presence of plaques, whereas paraoxonase 3 (PON-3) showed protective, anti-atherogenic properties. These findings illustrate how proteomic approaches can complement vascular imaging to uncover mechanisms and candidate biomarkers of cardiovascular comorbidity in axSpA.^101^ These studies highlight the growing body of evidence that supports the use of proteomic profiling and immune marker identification as key tools for understanding axSpA, offering novel avenues for diagnostic and therapeutic strategies.

**Figure 3.**
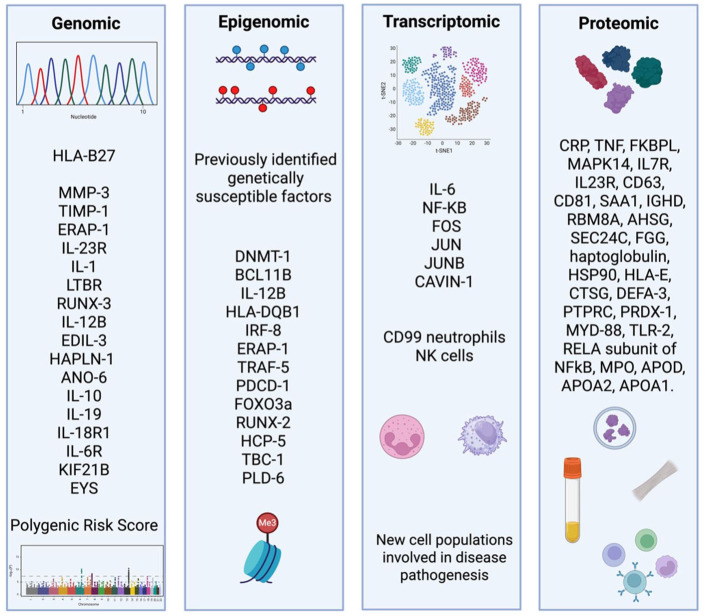
Overview of omics technologies applied to axSpA. Genomic studies have identified several susceptibility genes, including *HLA-B27, ERAP-1, IL-23R*, and others, with polygenic risk scores being explored for predictive purposes. Epigenomic approaches have highlighted DNA methylation changes and dysregulation of genes such as *DNMT-1, IRF-8*, and *TRAF-5*. Transcriptomic analyses reveal key inflammatory pathways (e.g., IL-17A, IL-6, NF-κB, FOS) and novel immune cell populations involved in disease pathogenesis, including CD4+ TRM17, CD99+ neutrophils, and NK cells. Proteomic profiling has uncovered numerous candidate proteins (e.g., CRP, TNF, IL-23R, HSP90, IL-6) that reflect inflammatory status and may support disease stratification. axSpA, axial spondyloarthritis; CD, cluster differentiation; CRP, C-reactive protein; DNMT-1, DNA methyltransferase 1; ERAP-1, endoplasmic reticulum aminopeptidase 1; FOS, Fos proto-oncogene; HLA, human leukocyte antigen; HSP90, heat shock protein 90; IL, interleukin; IRF-8, interferon regulatory factor 8; TNF, tumor necrosis factor; TRAF-5, TNF receptor associated factor 5; TRM17, tissue-resident memory Th17.

Taken together, high-throughput omics approaches encompassing genomics, epigenomics, transcriptomics, and proteomics have greatly advanced insights into the molecular pathways driving axSpA and have enabled the discovery of multiple candidate biomarkers (Figure 3).

## Conclusion and future perspectives

Significant advancements in imaging techniques and AI have contributed to improved diagnostic accuracy and earlier detection of axSpA, laying the groundwork for more standardized and refined disease assessment. AI-driven models have shown promising results in enhancing image interpretation, increasing diagnostic sensitivity, and reducing interobserver variability, particularly in complex or borderline cases. Importantly, several validation studies are already available in this field, supporting the robustness of these approaches. Although these technologies are not yet fully integrated into routine clinical workflows, their incorporation appears increasingly feasible in the near future, based on growing evidence.

Omics technologies, including genomics, epigenomics, transcriptomics, and proteomics, have expanded our understanding of the molecular mechanisms underlying axSpA and facilitated the identification of numerous potential biomarkers. Nonetheless, their translation into clinical practice remains limited. This gap is largely explained by the scarcity of large-scale validation studies, the lack of methodological harmonization, and the absence of regulatory approval pathways. To date, no omics-derived biomarker has been successfully implemented in routine clinical decision-making for axSpA. Most studies remain exploratory, and the few validation efforts reported so far are generally restricted to relatively small patient cohorts.

To accelerate clinical translation and align future research efforts, we propose the following priorities:

1. Validation: Conduct large, multicenter studies to validate promising biomarkers and AI-based tools across diverse populations, while overcoming current challenges such as the scarcity of samples and heterogeneity across patient cohorts.
2. Standardization: Harmonize data acquisition protocols, analytical methods, and reporting standards to ensure reproducibility and comparability, addressing persistent methodological challenges across different omics platforms.
3. Integration: Develop multi-omics models and AI-integrated pipelines that combine clinical, imaging, and molecular data for improved patient stratification.
4. Regulatory and clinical readiness: Promote early dialogue with regulatory bodies and clinicians to facilitate implementation of validated tools in real-world settings.

By addressing these key areas, the field can move closer to realizing the promise of precision medicine in axSpA. Differentiating between exploratory research and clinically actionable innovations will be essential to ensuring meaningful progress and improved patient outcomes.

## Limitations

This review has certain limitations. The studies included vary in design, population characteristics, and analytical approaches, which may contribute to some degree of heterogeneity. Nevertheless, most omics-based findings in axSpA are still in an exploratory phase, and continued validation in larger, longitudinal cohorts will be essential to facilitate their future clinical translation.

## Declarations

**Ethics approval and consent to participate:** Not applicable.

**Consent for publication:** Not applicable.

**Availability of data and materials:** Not applicable.
